# Validity and Reliability of Emergency Severity Index and Conventional Three-Tier Triage System in the Emergency Department, Hospital Universiti Sains Malaysia

**DOI:** 10.21315/mjms2020.27.2.10

**Published:** 2020-04-30

**Authors:** Ban Jin Victor Lim, Shaik Farid Ab Wahab, Yee Cheng Kueh

**Affiliations:** 1 Department of Emergency Medicine, School of Medical Sciences, Universiti Sains Malaysia, Kelantan, Malaysia; 2 Unit of Biostatistics and Research Methodology, School of Medical Sciences, Universiti Sains Malaysia, Kelantan, Malaysia

**Keywords:** emergency severity index, inter-rater agreement, sensitivity, specificity, triaging system

## Abstract

**Background:**

The study aimed to examine the reliability and validity of the existing three-tier triaging system and a new five-level emergency triaging system, emergency severity index (ESI), in the Emergency Department (ED) of Hospital Universiti Sains Malaysia (HUSM).

**Methods:**

This study was conducted in HUSM’s ED over two study periods. In the first three months, 300 patients were triaged under the three-tier triaging system, and, in the subsequent three months, 280 patients were triaged under the ESI. The patients were triaged by junior paramedics and the triage records were retained and later re-triaged by senior paramedics. The inter-rater reliability was evaluated using Cohen's Kappa statistics. The acuity ratings of the junior paramedics were compared with those of the expert panel to determine the sensitivity and specificity of each acuity level for both the ESI and the three-tier triaging system. The over-triage rate, under-triage rate, amount of resources used, admission rate and discharge rate were also determined.

**Results:**

The inter-rater agreement for the three-tier triaging system was 0.81 while that of the ESI was 0.75. The ESI had a higher average sensitivity of 74.3% and a specificity of 94.4% while the three-tier system’s average sensitivity was 68.5% and its specificity 87.0%. The average under-triage and over-triage rates for the ESI were 10.7% and 6.2%, respectively, which were lower than the three-tier system’s average under-triage rate of 13.1% and over-triage rate of 17.1%. The urgency levels of both the ESI and the three-tier system were associated with increased admission rates and resources used in the ED.

**Conclusion:**

The ESI’s inter-rater reliability was comparable to the three-tier triaging system and it demonstrated better validity than the existing three-tier system.

## Introduction

It is common in an Emergency Department (ED) to receive large numbers of patients that frequently overwhelms the personnel and resources available (1–2). A valid and reliable triage system is imperative to an ED in efficiently separating those severely ill patients from the crowds and to be triaged into critical zone where treatment and resuscitation work could be carried out in time. While under-triage compromises the patients’ health, over-triage causes unnecessary strain to human and physical resources (3–4). Frequently, inappropriate triaging is due to inexperienced triaging officer, complexity of patient’s illness and limited resources available at the particular time (5).

The ED in Hospital Universiti Sains Malaysia (HUSM) has been practicing three-tier triaging system since its establishment in year 1982. Similar triaging system is practiced by most ED in government and private hospitals in Malaysia. In three-tier triaging system, patients are categorised to three groups: i) Red zone – patients that are most ill and in critical condition; ii) Yellow zone – patients whose clinical condition are stable but requires some treatment and investigations to reach diagnosis; iii) Green zone – patients with most stable clinical condition and frequently can be managed as outpatient.

**Figure 1:**
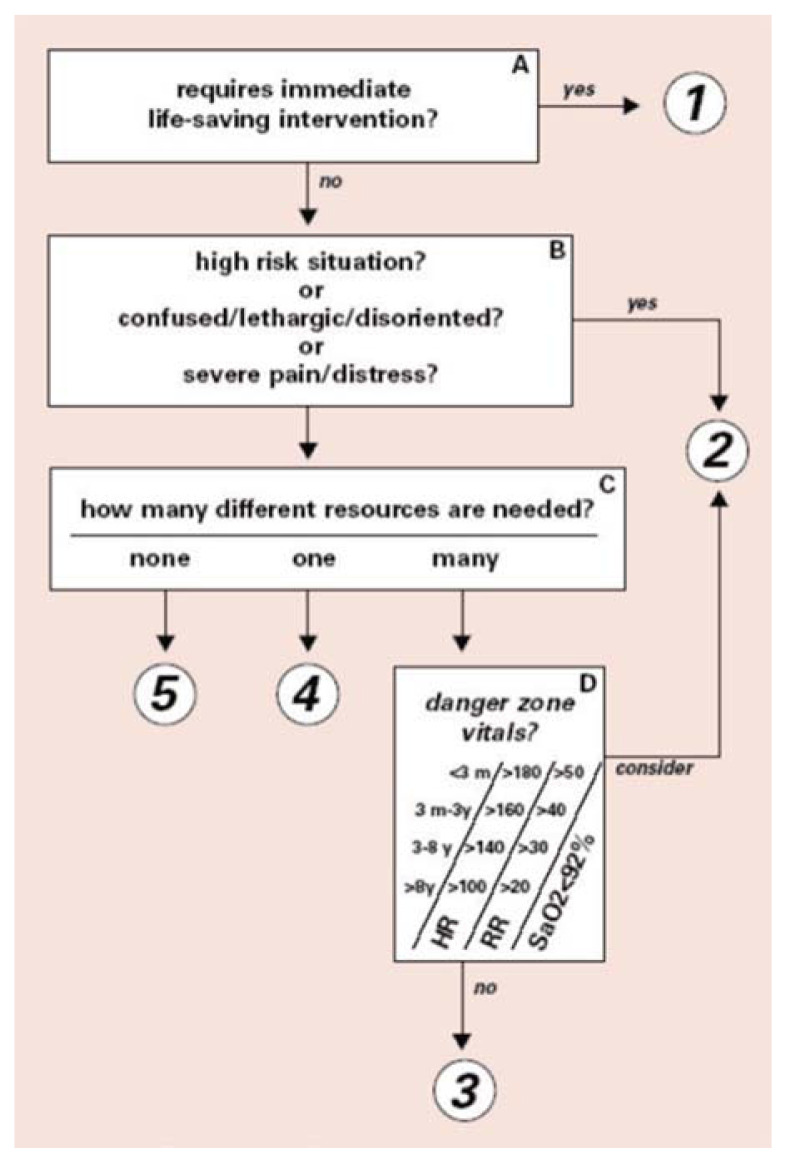
ESI triaging system algorithm

Emergency severity index (ESI) is a five-tier triaging system that was invented by Dr Richard Wuertz, an emergency physician in the United States in the late 1990s (6). It is a triaging system that triage all type of patients (trauma/non-trauma, pediatrics/adults/geriatrics) based on a single algorithm (Figure 1). Various papers have been published regarding ESI, citing its good inter-rater reliability and strong validity (7–9). The ESI has been extensively validated in a variety of populations and different countries around the globe (e.g. United States, Canada, Netherlands, United Kingdoms, South Korea, Taiwan, China, Australia, Middle East countries etc.) (5–23).

This was a first study in ED HUSM that was aimed to determine the inter-rater reliability and validity of the existing three-tier triaging system and ESI triaging system, and to descriptively compare both parameters in ED HUSM.

## Methods

### Participants and Procedure

This was a single-centered, cross-sectional, observational, comparative study between the conventional three-tier triaging system and the new five-tier ESI triaging system. The study was conducted at ED HUSM, a level one trauma centre with approximately 60,000 visits annually.

This study was conducted over two different study periods; i.e. three-tier triaging system from September 2016 until November 2016 and ESI triaging system from December 2016 until February 2017. Written consents have been taken from patients and paramedics that were enrolled in the study.

Before the study began, 10 junior paramedics (14.93% of total junior paramedics) who had less than five years working experience and five senior paramedics (21.74% of total senior paramedics) with at least five years of working experience were recruited for this study. The five years working experience was chosen as demarcation for senior and junior paramedics (27). The working experience of the paramedics was expected to be proportionate to the triage accuracy (24–26). They had underwent four hours or two sessions of classroom based ESI training and subsequently passed the test on different clinical scenarios for ESI triaging. This ESI training was to ensure that the raters (junior paramedics and senior paramedics) would understand fully on the new implementation of ESI triaging system. This could reduce the errors made by the raters due to misunderstanding on the new ESI triaging system.

Purposive sampling method was employed in this study. The first 30 patients were recruited every day on different shifts. For example, first day researcher recruited patients from the morning shift, second day from the afternoon shift, third day from the night shift, fourth day from the morning shift and the cycle repeats for subsequent day. For this study, walk-in patients were triaged by junior paramedics at the triage counter into different triage acuity ratings, i.e. Red/Yellow/Green or ESI1/ESI2/ESI3/ESI4/ESI5 by assessing their presenting complaints and physical condition. Patients aged younger than 18 years old or referred case from other hospitals/clinics or ambulance cases were excluded from this study.

Subsequently, the triage records of the patients were photocopied and reviewed by senior paramedics. The senior paramedics were blinded by the initial triage acuity ratings and would assign their own triage ratings based solely on the information written in the triage records. Triage records that were found incomplete were excluded from the study. Finally, the triage acuity ratings of the junior and senior paramedics were compared using Cohen’s Kappa statistic (10). The Kappa values or inter-rater reliability were counted for both three-level triaging system and five-level ESI triaging system.

As of today, there’s no gold standard in triaging patients (10–11). Thus, the reference standard used for this study are acuity ratings assigned by an expert panel which consists of the principal investigators (i.e. an emergency physician and an emergency registrar). Both principal investigators have vast experience working in an ED. The Emergency Physician is a senior consultant and lecturer in a public university and has more than 15 years of working and teaching experience. While the Emergency Registrar is a post-graduate student of the Department of Emergency Medicine who has eight years of working experience and is a certified Advanced Trauma Life Support (ATLS) and Advanced Cardiac Life Support (ACLS) provider. They are well-versed of the conventional three-tier triaging system (Malaysian triage categories) and they have passed ESI interactive web based training course. Two experts assessment was used as gold standard which was considered sufficient and adequate in the present study. This number of experts was also employed in other studies (11–12). Both had retrospectively and independently reviewed the patients’ ED record which includes the initial triage records, attending medical officer notes, investigations results and final diagnosis. By knowing the outcome of each patient, the expert panel would get a comprehensive picture of each patient’s condition and therefore, the acuity ratings assigned to each patient would be more befitting and accurate (11–12). They had discussed and reached a consensus in assigning acuity rating for each and every patient. The expert panel’s acuity ratings were used to compare with the junior paramedics’ acuity ratings to determine the sensitivity and specificity for both three-tier and ESI triaging systems (10, 12). Predictive values, percentage of agreement/under-triage/over-triage were also calculated. Case-mix measurements such as hospital admission rate (patient admitted to either observational ward or hospital ward), discharge rate (patient discharged from ED) and amount of resources used in ED were also determined as part of validity study for both three-tier and ESI triaging system (8, 11, 13).

### Data Collection and Definitions

The following data were extracted from patients’ ED records: gender, ethnic, age, number of co-morbidities, initial triage ratings by junior paramedics, triage rating by senior paramedics, triage ratings by expert panel, patients’ disposition (i.e. admission which is defined as patient admitted to either observational ward or hospital ward or patient requested discharge at-own-risk or discharge) and amount of resources used in ED, which is defined according to ESI definitions (Table 1). Resources used included laboratory investigations, radiology imaging, electrocardiograms, intra-muscular or intravenous medication, intravenous fluids, primary team or specialty consultation and procedures done on patient.

### Data Analysis

Data analysis was performed by using IBM SPSS version 24. The reliability of both three-tier and ESI triaging system were measured using Cohen’s Kappa statistic by comparing the junior paramedics triage ratings with senior paramedics triage ratings. The validity of both triage system were determined by comparing junior paramedics triage ratings to reference standard, i.e. expert panel triage ratings. Sensitivity and specificity, predictive values, agreement percentage, under and over-triage percentage, hospital admission rate, discharge rate and amount of resources used in ED were also computed.

Descriptive statistics with continuous data are presented as mean with standard deviation (SD), whereas categorical data are presented as frequency and percentage with *P*-value < 0.05 indicates a statistical significant difference.

## Results

During first study period, a total of 300 patients were triaged under the three-tier triaging system. However, only 280 usable samples (i.e. data required completed, 1.55% of total patients) were included in the study. At second study period, 280 patients were triaged under the ESI triaging system and only 262 usable samples (1.45% of total patients) were included in the study. The omitted samples were due to incomplete triage record by junior paramedics or illegible triage paramedics’ signature.

The patients’ characteristics for both ESI and three-tier triaging were analysed according to gender, age, ethnics, type of cases and number of co-morbidity in a patient were shown in Table 2.

The acuity ratings by junior paramedics were compared with senior paramedics for both triaging system by using Cohen’s Kappa statistics as shown in Tables 3 and 4. The agreement for three-tier triaging system was 0.81 with standard error of 0.04 while the agreement for ESI triaging system was 0.75 with standard error of 0.03. Both triaging system showed good strength of agreements (14).

The sensitivity and specificity, and predictive values for both three-tier and ESI triaging system are shown in Table 5.

**Figure 2:**
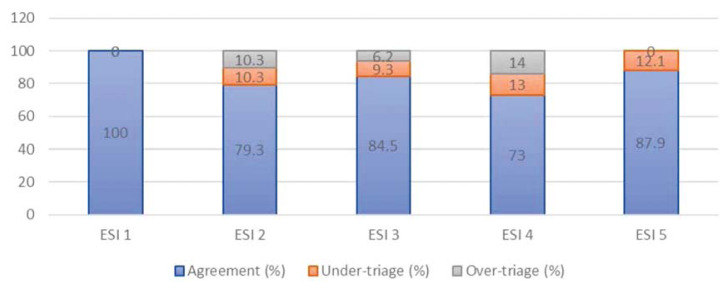
Agreement of acuity ratings between junior paramedics and expert panel for ESI

**Figure 3:**
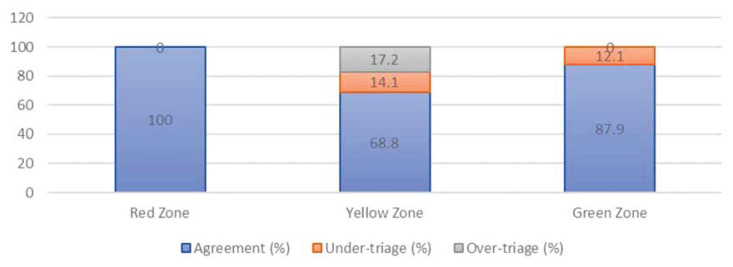
Agreement of acuity ratings between junior paramedics and expert panel for three-tier triaging system

The agreement/under-triage/over-triage rate for both ESI and three-tier triaging system are shown in Figures 2 and 3, respectively.

**Figure 4:**
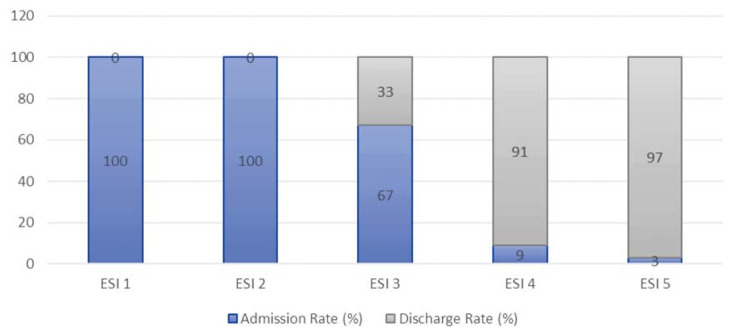
Hospital admission rate and discharge rate for ESI triaging system

**Figure 5:**
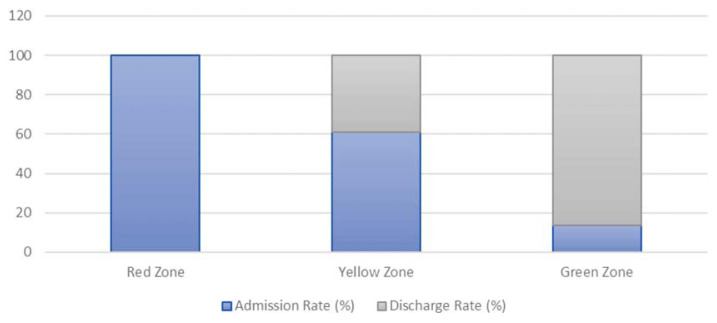
Hospital admission and discharge rate for three-tier triaging system

The admission and discharge rate for each acuity ratings for both ESI and three-tier triaging system are shown in Figures 4 and 5.

The amount of resources used in ED was strongly associated with urgency level for both ESI and three-tier triaging system, meaning the higher the acuity rating, the more resources were consumed. The number of resources used in ED was represented as mean with standard deviation and are shown in Table 6.

## Discussion

This was the first head-to-head comparison study of a five-tier ESI triaging system with conventional three-tier triaging system that has been used in ED HUSM since its establishment in 1990s. The patients’ profile for both triaging system was identical and comparable. In terms of gender, male and female patients were almost equal in number. Malays remain the main ethnic that visited the ED. Majority of the patients in ED had none or one co-morbidity. Non-trauma cases remained the bulk of ED cases.

Both ESI and three-tier triaging system had substantially good inter-rater agreement (i.e. in Cohen’s Kappa statistic: 0.61–0.80 signifies substantial agreement) for triage acuity ratings between junior and senior paramedics. Three-tier triaging system with weighted Cohen’s Kappa of 0.81 had slightly outperformed ESI with weighted Cohen’s Kappa 0.75. The result was understandable given three-tier triaging system had been in place for many years and the paramedics were more well-versed and accustomed in the existing three-tier triaging system (15–16). The paramedics only underwent two classes or four hours of ESI training prior to the study. In another way, it could be seen that at least the new ESI triaging system was noninferior to three-tier triaging system in terms of inter-rater agreement. In this study, the inter-reliability of both three-tier and ESI were higher compared to study done by Travers et al which were 0.53 for three-tier and 0.68 for ESI (10).

In terms of sensitivity and specificity, ESI had outperformed three-tier triaging system. ESI had average of sensitivity of 74.3% and specificity of 94.4% compared to three-tier sensitivity of 68.5% and specificity of 87.0%. The positive predictive values (PPV) of ESI and three-tier almost identical with 85.1 and 85.4, respectively and ESI had better negative predictive values (NPV) of 94.9 to three-tier 89.9. These results showed that ESI was a much superior triaging tool than three-tier triaging system. These results were identical to other studies and the main reason for ESI being more accurate and precise was due to its explicitly written criteria in ESI triaging algorithm (10–12, 17). ESI was more accurate in terms of identifying severely ill patients and this was vital as it ensured that these patients received treatment in time (18). At the same time, ESI was a safer triaging tool in identifying those relatively stable patients and segregated them according to their resources need (13, 18–19).

Both ESI and three-tier triaging systems had 100% agreement with the expert panel critically ill patient (i.e., ESI 1, ESI 2 and Red zone). This result was expected and was similar to other studies (20–21) as these patients usually came with unstable vital signs and were clearly indicated to be triaged to the most critical zone whereby resuscitation work were carried out immediately. In terms of triaging relatively stable patients but need complex evaluation prior to admission or discharge (ESI 3 or Yellow zone patients), ESI again outperformed three-tier triaging system). ESI 3 had 9.3% of under-triaged cases and 6.2% of over-triaged cases compared to three-tier 14.1% under-triaged and 17.1% over-triaged. Under-triage compromised patients’ care whereas over-triage put unnecessary stress to human and physical resources in ED (1–2). Thus, ESI appeared to be a safer and more efficient triaging tool. The under-triaged cases for most stable patient for ESI was identical to three-tier triaging system at 12.0%, respectively.

ESI and three-tier shared similar traits in terms of hospital admission/discharge rate. Those severely ill patients especially at ESI 1, ESI 2 and Red zone patients were 100% admitted. Whereas those stable but needed complex investigations patients such as ESI 3 or Yellow zone patients had more admission rate (67% for ESI 3, 60% for Yellow zone) than discharge rate. As for the most stable patients such as ESI 4, ESI 5 and Green zone patients, most of them were discharged with 90% discharge rate for each category. The admission rate was proportional to the urgency level for both triaging systems and similar result was seen in other studies (10, 20, 22–23).

The patterns of consumption of ED resources were identical for both ESI and three-tier triage system. The more severe the patient, the more resources he or she consumed and the result was comparable to other studies (7–8, 13, 19, 22). The mean resources used in ED for critically patients like in ESI 1 was 6.00, ESI 2 was 4.76 and Red zone was 5.22. The mean resources used in ED for stable patients that were triaged to ESI 4 was 1.15, ESI 5 was 0.30 and Green zone was 1.29. The main advantage of ESI is its ability to segregate most stable patients into another two subgroups, namely ESI 4 and ESI 5 based on patients resources requirements. Thus, it is possible for ED to form a fast-track lane to treat this group of patient and relieve the congestion in ED and at the same time increase overall satisfaction among patients (20–23).

## Limitations

The main limitation for this study was the senior paramedics could only retrospectively reviewed the photocopies of triage record written by junior paramedics. Their triage decision were limited by the information contained in the triage record and could not assess the patients physical condition and could not ask relevant questions to the patients. This limitation was unavoidable as the idea of putting one senior and one junior paramedics behind the triage counter and triage the patients independently was simply not feasible. Firstly, the whole triaging process would not be efficient if a same patient was triaged twice by the junior and the senior paramedics. Secondly, if ultimately the patient would always follow the triage acuity ratings given by the senior paramedics then there would be tendency for junior paramedics to follow suit the senior’s triage ratings.

The second limitation of this study was the paramedics were still relatively unfamiliar with the new ESI triaging system. They had only underwent four hours of ESI classes and passed the written scenarios exam prior to triage patient in real time. Ideally, the paramedics were given a period of adaptation (about two months) of triaging real patients with ESI before they start triaging patient for the study.

## Conclusion

This study has shown ESI is a superior triaging tool than three-tier triaging system. The ESI had inter-rater reliability that was comparable to the three-level triaging system and had better sensitivity and specificity than the existing three-tier triaging system. ESI could be the future triaging tool for the ED. However, one needs ESI regular re-training or re-teaching, for example two-monthly ESI training course to create ESI proficiency among paramedics to ensure consistency or improvement in triaging accuracy.

## Figures and Tables

**Table 1:** ESI defination of resources and non-resources

Resources	Non-resources
Labs (blood, urine)	History taking
ECG	Physical examination
	Point-of-care testing (reflo, PEFR)
Imaging: X-rays, CT, MRI	Prescription refills
IV fluids	Saline or heparin lock
IV or IM or Neb medications	PO medications
	IM tetanus injection
Specialty consultation	
Simple procedure = 1	Simple wound care (check, dressing)
(simple T&S, foley catheter, STO)	Crutches, splinting
Complex procedure = 2	
(complicated T&S, procedural sedation analgesia)	

Notes: ECG = electrocardiogram; CT = computed tomography; MRI = magnetic resonance imaging; IV = intravenous; IM = myocardial infarction; T&S = type and screening; STO = suture-to-off; PEFR = peak expiratory rate flow

**Table 2:** Patient characteristics

	ESI (*n* = 262)	Three-tier (*n* = 280)
Sex (%)		
Male	145 (55.3)	137 (48.9)
Female	117 (44.7)	143 (51.1)
Age, year		
Mean (+/− SD)	41.10 (19.30)	40.33 (18.41)
Type of cases (%)		
Trauma	41 (15.7)	54 (19.3)
Non-trauma	221 (84.3)	226 (80.7)
No. of co-morbidity(s) in a patient (%)		
0	150 (57.2)	170 (60.7)
1	55 (21.0)	58 (20.7)
2	26 (10.0)	31 (11.1)
3	22 (8.4)	16 (5.7)
4	9 (3.4)	5 (1.8)
Ethnics (%)		
Malay	251 (95.8)	255 (91.1)
Chinese	8 (3.0)	13 (4.6)
Indian	2 (0.8)	8 (2.9)
Others	1 (0.4)	4 (1.4)

**Table 3:** Inter-rater reliability of ESI

ESI 1		Junior triage officer					Total

		ESI 2	ESI 3	ESI 4	ESI 5		
Senior triage officer	ESI 1	4	0	0	0	0	4
	ESI 2	0	25	8	0	0	33
	ESI 3	0	4	83	18	0	105
	ESI 4	0	0	6	80	7	93
	ESI 5	0	0	0	2	25	27

Total		4	29	97	100	32	262

Note: Weighted Kappa = 0.75 with standard error: 0.03

**Table 4:** Inter-rater reliability of three-tier triage system

Red		Junior triage officer			Total

		Yellow	Green		
Senior triage officer	Red	7	1	0	8
	Yellow	2	54	10	66
	Green	1	8	197	206

Total		10	63	207	280

Note: Weighted Kappa = 0.81 with standard error: 0.04

**Table 5:** Validity of ESI and three–tier triaging system

	Sensitivity	Specificity	PPV	NPV
ESI 1	57.1	100	100	98.8
ESI 2	71.9	97.4	79.3	96.1
ESI 3	84.5	90.9	84.5	90.9
ESI 4	89.2	85.5	74.0	94.4
ESI 5	69.0	98.2	87.9	94.3

Average	74.3	94.4	85.1	94.9

Red	47.4	100	100	96.3
Yellow	63.8	90.5	68.8	88.4
Green	94.3	70.5	87.4	84.9

Average	68.5	87.0	85.4	89.9

**Table 6:** Amount of resources used in ED for ESI and three-tier triaging systems

Triaging systems	Mean	SD
ESI 1	6.00	0
ESI 2	4.76	0.786
ESI 3	3.29	1.172
ESI 4	1.15	0.903
ESI 5	0.30	0.951
Red zone	5.22	0.667
Yellow zone	3.39	0.619
Green zone	1.29	1.187
